# pUC18-CpG Is an Effective Adjuvant for a Duck Tembusu Virus Inactivated Vaccine

**DOI:** 10.3390/v12020238

**Published:** 2020-02-20

**Authors:** Xiao Ren, Xiaolei Wang, Shan Zhang, Xintao Gao, Lichun Fang, Xixi Wang, Weidong Lin, Hong Jia, Xiaoyu Guo, Ting Xin, Hongfei Zhu, Jian Lin, Shaohua Hou

**Affiliations:** 1 Institute of Animal Sciences, Chinese Academy of Agricultural Sciences, Beijing 100193, China; rxbioclub@sina.com (X.R.); zhangshan0276@163.com (S.Z.); 18854881003@163.com (L.F.); wangxixinl1990@126.com (X.W.); lwdong21@163.com (W.L.); jiahong@caas.cn (H.J.); vetiascaas@126.com (X.G.); xinting_xt@163.com (T.X.); bioclub@vip.sina.com (H.Z.); 2 Institute of Animal Husbandry and Veterinary medicine, Beijing Academy of Agriculture and Forestry Sciences, Beijing 100193, China; wangxl227@126.com; 3 Biotechnology Research Institute, Chinese Academy of Agricultural Sciences, Beijing 100081, China; xintao.gao@hotmail.com

**Keywords:** pUC18-CpG, adjuvant, duck Tembusu virus, inactivated vaccine

## Abstract

Duck Tembusu virus (DTMUV) is an emerging pathogenic flavivirus responsible for massive economic losses in the duck industry. However, commercially inactivated DTMUV vaccines have been ineffective at inducing protective immunity in ducks. The widely used adjuvant cytosine-phosphate-guanine oligodeoxynucleotides (CpG ODNs) reportedly improve humoral and cellular immunities in animal models. However, its effectiveness in DTMUV vaccines requires validation. Here, we assessed the protective efficacy of pUC18-CpG as an adjuvant in an inactivated live DTMUV vaccine in ducks. Our results revealed that the serum hemagglutination inhibition (HI) antibody titers, positive rates of anti-DTMUV antibodies, the concentration of serum cytokines, and protection efficacy were significantly increased in ducks immunized with pUC18-CpG compared to that in the control group. Moreover, ducks immunized with a full vaccine dose containing a half dose of antigen supplemented with 40 μg of pUC18-CpG exhibited the most potent responses. This study suggests that pUC18-CpG is a promising adjuvant against DTMUV, which might prove effective in treating other viral diseases in waterfowl.

## 1. Introduction

In 2010, a newly emerging infectious disease in ducks broke out in many Chinese coastal provinces. The disease is characterized by a substantial drop in egg-laying and low mortality [1]. The causative agent has been identified as duck Tembusu virus (DTMUV), an enveloped positive sense single-stranded RNA virus that belongs to the genus Flavivirus, family Flaviviridae [2,3,4]. DTMUV, similar to other flaviviruses, is a mosquito-borne Flavivirus, and was first isolated from mosquitoes of the genus Culex in 1970s in Malaysia [5].

Since its emergence, the Tembusu virus infection has led to major economic losses in the Chinese poultry industry [6]. Nearly all duck species have been reported to carry the DTMUV, including Cherry Valley ducks, Pekin ducks, and shelducks [7]. Further, this virus is efficient at infecting other animal species, including chickens [8,9], geese [10,11], mice [12], pigeons [1], and sparrows [13], thus indicating that DTMUV has an extensive host range. A recent report has demonstrated that DTMUV can also infect humans [14]. Therefore, DTMUV can pose a severe economic burden on the poultry industry while simultaneously constituting a public health threat.

Although an effective attenuated DTMUV vaccine candidate has been reported [15], immunization with this type of vaccine hampers the differentiation of infected and vaccinated ducks based on serology. In addition, the cost of attenuated vaccines is prohibitive, and improper administration may cause potential virulence reversion [16]. Alternatively, an inactivated live DTMUV vaccine is available; however, it has been shown to induce low protective antibody titers in ducks and, thus, is not completely effective in protecting immunized ducks [17]. A more cost-effective and safer DTMUV vaccine variant is, therefore, urgently needed to tackle this emergent disease and assist the duck industry.

The bacterial genomic DNA, or synthetic unmethylated cytosine-phosphate-guanine oligodeoxynucleotides (CpG ODNs), which contain bioactive CpG motifs, elicit immunostimulatory effects and are considered safe and effective immunopotentiator adjuvants. These molecules reportedly enhance humoral and cellular immunity in humans and animals [18,19,20]. It has now been recognized that the human immune system, similar to other vertebrates, identifies unmethylated CpG dinucleotides in specific situations involving dangerous stimuli or infection, thereby activating immune response cascades [21]. Moreover, studies have shown that when the exogenous CpG motif is inserted into an empty plasmid vector (pUC-18), it significantly enhances the immune response to a vaccine [22,23]. To the best of our knowledge, CpG ODNs have preliminarily been explored as immunostimulants or adjuvants in waterfowl (including ducks), and satisfactory results have been achieved [24,25].

This study evaluated the adjuvant potential of pUC18-CpG in an inactivated DTMUV (HB strain) vaccine administered to ducks intramuscularly. Our analyses examined the effects of the vaccine on several immune parameters, including the titer of serum hemagglutination inhibition (HI) antibodies, post-immunization protein expression levels of immune responses-related genes, and post-challenge protection efficacy.

## 2. Materials and Methods

### 2.1. Virus Preparation

The Tembusu-HB viral strain used in this study was isolated, identified, and provided by the Institute of Animal Husbandry and Veterinary Medicine at the Beijing Academy of Agricultural and Forestry Sciences. The embryos and allantoic fluid were collected from in 10.5-day-old specific pathogen-free (SPF) duck embryos, previously inoculated with the Tembusu-HB virus strain. The EID_50_ (50% embryo infection dose) of DTMUV-HB was detected to be 10^7.5^/mL.

### 2.2. pUC18-CpG

The CpG-enriched pUC18 containing 20 copies of CpG ODN 2006 (sequence: 5′-TCGTCGTTTTGTCGTTTTGTCGTT-3′) was generated by tandem insertion into the MCS of the pUC-18 vector. pUC18-CpG was propagated in *Escherichia coli*, then cultured by fermentation, and the recombinant plasmid was purified using a large-scale plasmid purification method [26]. Subsequently, *Limulus amebocyte* lysate tests were carried out to verify the removal of endotoxin. The CpG motif and copy number in the plasmid were confirmed by sequencing. The purified plasmid was dissolved in endotoxin-free phosphate-buffered saline (PBS) to a concentration of 2 mg/mL and stored at −80 °C.

### 2.3. Animals

Pekin ducks, bought from the Beijing Nankou Pekin Duck Breeding Center (Beijing, China), were seronegative for DTMUV antibodies. SPF duck eggs were purchased from the Laboratory Animal Center at the Harbin Veterinary Research Institute, Chinese Academy of Agricultural Sciences (Harbin, China). The 6-day-old SPF chicken embryos were obtained from Beijing Boehringer Ingelheim Vital Biotechnology Co., Ltd. (Beijing, China). A total of 104 healthy 42-day-old ducks were selected and randomly assigned to groups for the vaccine trial and challenge test. The in vivo experimental procedures were approved by the Animal Care and Use Committee at the Institute of Animal Sciences from the Chinese Academy of Agricultural Sciences, China (IACUC.No.PJ.2011-012-03).

### 2.4. Vaccine Preparation

The vaccines were prepared according to the production standards established by Ringpu Biological Pharmaceutical Co., Ltd. (Baoding, China). The allantoic fluid was inactivated by adding formaldehyde to a final concentration of 0.5% and mixing thoroughly, followed by incubating at 37 °C for 48 h. The vaccines were created by combining allantoic fluid containing inactivated DTMUV-HB with a sterilized mixture containing 94% (*v*/*v*) Marcol 52, 6% (*v*/*v*) span-80, and 2% (*w*/*v*) aluminum stearate. Control vaccines were prepared similarly using allantoic fluid from duck embryos inoculated with PBS. pUC18-CpG was added as the adjuvant to the inactivated oil vaccines. The vaccine formulations are presented in Table 1.

One-hundred-and-four healthy 42-day-old ducks were randomly divided into groups and intramuscularly (IM) administered with inactivated vaccine, vaccines containing CpG ODNs, or the negative control containing PBS. The detailed experimental design is presented in Table 2. Briefly, ducks were boosted on day 14 (D14) following the primary immunization. Blood samples were collected before vaccination (D0), as well as on D14, D24, D35, and D56 after primary vaccination.

### 2.5. Quantification of Serum Antibody Titers via HI Assays

Serum was isolated from post-vaccination blood samples, and antibody titer quantitation was conducted using a modified HI assay. Briefly, DTMUV infected allantoic fluid was inactivated by beta-propiolactone, and viral antigens were filtrated for HI assays. The unit of HA antigen in each preparation was determined by a hemagglutination (HA) test, which is defined as the reciprocal of the highest dilution of purified antigen that can still cause complete hemagglutination. The antigen was diluted with 0.4% bovine serum albumin borate sodium (BABS) chloride solution, containing 4 HA units per 25 μL. The test serum samples were serially diluted (1:2) in U-bottomed 96-well plates. Four units of HA antigen were added, and the mixture was incubated overnight at 4 °C. Then, 50 µL 0.33% goose red blood cell suspension was added to each well. The plates were incubated for 1 h at 37 °C. DTMUV positive and negative serum controls, as well as a goose erythrocyte control, were included in each plate. The serum HI titers were expressed as the reciprocal of the highest serum dilution that demonstrated complete inhibition of hemagglutination. Positive titers were interpreted as inhibition of hemagglutination at a serum dilution of 1:20 or higher. The positive rates of antibody and the geometric mean titer (GMT) for each group were further determined, and statistical analysis was performed. The GMT is less affected by extreme values than the mean HI titer, which can reduce the influence of individual differences, and is more suitable to reflect the average level of the whole group. Moreover, GMT is a recommended standard statistic for summarizing HI titers [27,28]. The GMT was quantified using the formula, GMT = lg^−1^[(N1 × lgX1 + N2 × lgX2 + … + Nn lgXn)/(N1 + N2 + … + Nn)] (N: number of ducks; X: the value of HI titer; lg^−1^(X) = 10^x^).

### 2.6. Determination of Serum Cytokine Levels

To evaluate changes in cytokine expression following vaccination, the concentrations of interferon-alpha (IFN-α), interferon-gamma (IFN-γ), interleukin-2 (IL-2), and interleukin-6 (IL-6) were quantified in serum samples from groups A1, B1, E1, and G1 at different time points using standard, cytokine-capture sandwich ELISA kits (Huabo Deyi, Beijing, China), according to manufacturer’s instructions. Briefly, diluted standards and serum samples (1:4) were added to microtiter plates coated with corresponding monoclonal antibodies. HRP-conjugated goat anti-duck IgG was used to detect bound antibodies, and the optical density (OD) was measured at 450 nm. The concentrations of cytokines were calculated using standard curves.

### 2.7. Virus Challenge

On D42, after boost immunization, six ducks randomly selected from each group were intramuscularly challenged with 100 DID_50_ (50% duck infection dose) of DTMUV-HB in a volume of 0.5 mL. Previous studies have observed that the Tembusu virus can be propagated in both chicken and duck embryos, and causes death typically 60 h after virus inoculation [17]. Blood was collected from the ducks on D2 post-challenge, and the resulting serum was inoculated into five SPF chicken embryos that were 6-days-old for virus isolation. Between 24–72 h after viral inoculation, embryo death was monitored and recorded, and the PD_50_ (50% protection dose) for each vaccine batch was determined using the Spearman–Kärber method [29].

### 2.8. Virus Isolation and RT-PCR

Virus isolation was further confirmed by a flavivirus-specific reverse transcriptase PCR (RT-PCR). Briefly, RNA was extracted from the deceased embryos using MiniBEST Universal RNA Extraction Kit (Takara, Beijing, China), according to manufacturer’s protocols. Two micrograms of total RNA from each sample were reverse transcribed into cDNA using PrimeScript™ RT Master Mix (Takara, Beijing, China), according to the manufacturer’s instructions. The detection primers were designed according to conserved DTMUV *NS5* gene sequences [30]: forward: 5′-TCAAGGAACTCCACATGA-3′; reverse: 5′-GTGTCCCATCCTGCTGTGTCATCAGCATACA-3′. The expected length of the amplified fragment was 998 bp, and the specific gene products were visualized on a 1% agarose gel.

### 2.9. Statistical Analysis

Statistical analysis was performed using GraphPad Prism 6 software (GraphPad Software, Inc., San Diego, CA, USA). Differences within each treatment at various time points were analyzed using two-way ANOVA. Data is shown graphically as the geometric mean of the fold change plus the standard error of the mean (SEM). A *p*-value < 0.05 was considered significant.

## 3. Results

### 3.1. pUC18-CpG Enhanced Serum Antibody Responses

**Figure 1:**
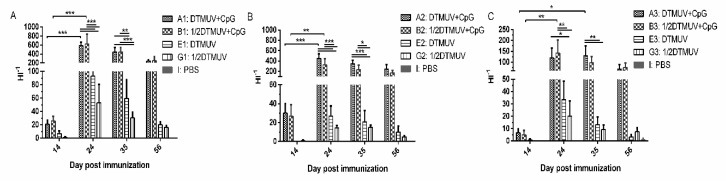
Serum hemagglutination inhibition (HI) levels in vaccinated ducks. Serum samples were collected from ducks vaccinated with (**A**) full, (**B**) 1/2, and (**C**) 1/4 doses at different time posts for HI antibody level. Antibody titers were determined using the HI assay with 4 HA units of the DTMUV-HB. The HI titer is expressed as the reciprocal form. Data are expressed as means ± SEM. (* *p* < 0.05, ** *p* < 0.01, *** *p* < 0.001).

Serum from vaccinated ducks was subjected to quantify the HI titers of the test groups. The details of results are summarized in Supplementary Material Table S1. No detectable antibody responses were obtained in any of the control ducks during the whole immunization. Note that the titer of HI antibody in groups A and B improved significantly during 14–24 dpi (day post-immunization) (except for HI titer in group A3, which improved during 14–35 dpi), and was significantly higher than that in group E and group G at 24 dpi and 35 dpi at any dose (Figure 1). The HI antibody titer increased with increasing dose but the difference was not statistically significant except for the comparison of 0.5–0.125 pair at 35 dpi (Supplementary Material Figure S1).

**Figure 2:**
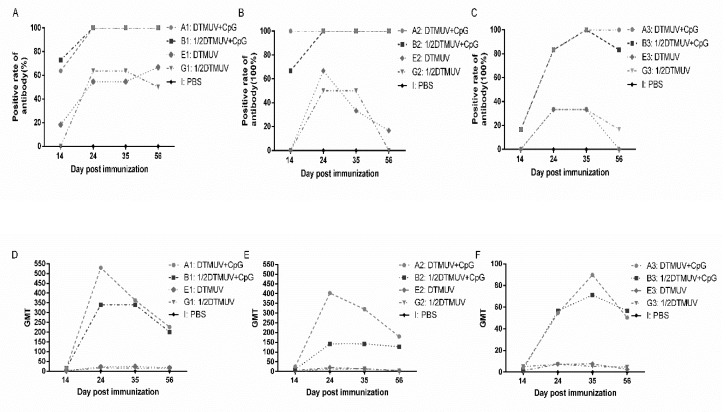
The positive rate of antibody and geometrical mean titer in each group immunized with (**A**,**D**) full, (**B**,**E**) 1/2, and (**C**,**F**) 1/4 doses of vaccines. Positive titers were interpreted as inhibition of hemagglutination at a serum dilution of 1:20 or greater.

We further implemented the statistical analysis of the positive antibody rates and GMT, as shown in Table S1 and Figure 2. The positive rates of group A and group B under different doses showed a similar trend during the experiment, reaching 100% and remaining at the same level for subsequent periods (except for positive rates of group B3, which declined after reaching 100% at 35 dpi). The positive rates of group A and group B at each time point were much higher than that of group E and group G. The positive rates of group E and group G were basically the same, and the positive rates decreased with the decrease of dose. The GMT trend of group A was inverted V-shaped with a peak at 24 dpi at full and 1/2 doses/at 35 dpi at a 1/4 dose. GMT in group B showed a plateau (24 dpi–35 dpi) at full and 1/2 doses and then decreased, whereas at a 1/4 dose, the trend of group B was similar to that of group A3. The GMT relationship among the four groups was A > B > E > G, and the GMT values of group A and B were much higher than those of group E and G.

### 3.2. pUC18-CpG Enhanced both Th1- and Th2-Type Cytokine Production

**Figure 3:**
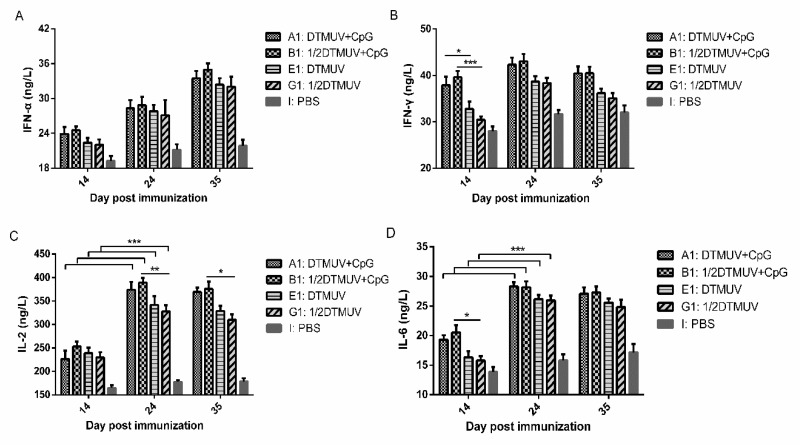
Cytokine protein production in response to vaccines. Ducks were immunized I.M. twice (D0 and D14) with a full dose of vaccines formulated with pUC18-CpG or not and ducks in the control groups were immunized with PBS. The protein expression of (**A**) IFN-α, (**B**) IFN-γ, (**C**) IL-2, and (**D**) IL-6, was detected by ELISA. Data are presented as the mean ± SEM of ducks in the same treatment. (* *p* < 0.05, ** *p* < 0.01, *** *p* < 0.001).

To identify potential immunological correlates with protection, we evaluated changes in the expression of specific proteins, including IFN-α (Figure 3A), IFN-γ (Figure 3B), IL-2 (Figure 3C), and IL-6 (Figure 3D), in response to vaccination, via ELISA. Ducks vaccinated with a full vaccine dose (A1, B1, E1, G1) were selected for this assay. As shown in Figure 3, the expressions of 3 of the 4 proteins were found to be upregulated in the serum of the groups immunized with pUC18-CpG compared to those immunized without the adjuvant. Specifically, the protein expression of IFN-γ (*p* < 0.001) and IL-6 (*p* < 0.05) in group B1 were significantly higher than those in group G1 at 14 dpi, whereas significant difference was observed in pair-comparison of group A1 and E1 for IFN-γ expression (*p* < 0.05) at that time point. Moreover, IL-2 expression level in group B1 was significantly higher (*p* < 0.01; *p* < 0.05) than that in group G1 at 24 dpi and 35 dpi. However, no significant differences were observed in IFN-α production among time points.

### 3.3. pUC18-CpG Enhanced Protection Efficacy

To evaluate the protective efficacy of the vaccines, the ducks were challenged with DTMUV-HB, and serum samples were collected after challenge. The control ducks exhibited clinical signs, such as green watery diarrhea, reduced feed intake, and depression. The RT-PCR data are consistent with the virus isolation results.

The detailed results of virus isolation in each duck, along with the respective protective rates and PD_50_ for each group are presented in Table S1 and Table 3. One-hundred percent of ducks in group A and group B receiving full and 1/2 doses of the vaccines with pUC18-CpG were protected against the virulent virus challenge. However, only 50% and 66.7% of ducks in the A3 and B3 groups, which received a 1/4 dose, were found to be protected, respectively. Furthermore, the PD_50_ values for group A and group B were determined to be 4.0 and 4.5, respectively. The protective rates of group E1 and group G1 were both 66.7% at a full dose, and decreased with the dose reduction. The PD_50_ value of group E was found to be only 1.4, which was lower than the 1.8 in group G.

## 4. Discussion

Duck Tembusu virus disease, an acute infectious disease caused by DTMUV [31], has not only caused significant economic losses in the poultry industry, but has also posed potential threats to public health [14,32]. Vaccination has proven effective in protecting ducks against DTMUV infection and is being used in specific areas to control the disease [17]. However, the ducks receiving an inactivated vaccine gradually developed antibodies yet exhibited low and non-protective antibody titers, highlighting the urgency of an effective vaccine.

CpG ODN is a standard adjuvant, employed in previous studies, that effectively enhances the protective efficacy of vaccines [33,34]. However, due to the high cost of synthetic CpG ODNs, it is not conducive to large-scale production and application. Within the field of DNA vaccines, the inclusion of antibiotic resistance genes in vector plasmids has been controversial. Several groups have opted to use different antibiotic-free selection systems to circumvent the potential antimicrobial resistance [35,36,37], but the technology was not yet mature and major deficiencies in these strategies cannot be addressed could not be addressed (such as the negative effect of increasing the number of plasmid copies on the bacteria growth during fermentation, the harm to humans caused by plasmids carrying redundant bacterial genes, high costs, unavailable large-scale production, etc.) [38,39,40], and no plasmid system has yet been approved for industrial mass production. In addition, antibiotics are added to feed during current breeding processes, making it difficult to evaluate in practice. Moreover, insertion of CpG motifs into the pUC-18 plasmid vector has also been proven to enhance the immune effect of vaccines [22,23]. In view of these, we finally circumvented the antibiotic-free selection system and chose the pUC18 plasmid vector. The large-scale fermentation technique in *E. coli* in our laboratory is mature, with the most production and lowest cost.

We utilized the pUC-18 vector containing CpG ODN-2006 as an adjuvant to generate a batch of vaccines for our study. Following vaccination, in pair-comparison of core vaccine groups, DTMUV vaccine (group E) generated higher values of antibody-related parameters and cytokine expression than 1/2 DTMUV vaccine (group G) at almost all time points and immunization doses. There are two possible reasons: one is that the inactivated DTMUV vaccines were not able to elicit an adequate immune response, another is that the generation of anti-DTMUV antibody may be antigen dose-dependent within a certain range.

When pUC18-CpG was added to vaccines, not only did more ducks show early immune responses at 14 dpi, but also a significantly improved HI titer (*p* < 0.001) during 14 dpi–24 dpi and a longer-term enhanced antibody response were observed in group A and B. In addition, the HI titer, antibody-positive rates, and GMT were higher than core vaccine groups (group E and group G) at all time points, and the difference was significant in HI titer between group A/B and group E/G (Table S1, Figure 1 and Figure 2). During the experiment, the average antibody level of group A was higher than that of group B at any dose while the protein expression level of cytokines in group B was found to be higher than that of group A when a 0.5 mL dose was administered, but the differences were not statistically significant (*p* > 0.05). The possible reason for the similar effect between group A and group B may be that in the case of pUC18-CpG addition, both the efficiency of antigen utilization and the level of immune response are all enhanced, and excessive antigen means more non-structural proteins, which may cause side effect.

It has become increasingly apparent that the IFN-γ-dependent Th1 cellular responses are required to elicit protection against Flavivirus infection [41,42]. Similarly, we found that ducks immunized with pUC18-CpG exhibited significantly higher levels of IFN-γ compared to those vaccinated without the adjuvant at 14 dpi (Figure 3), promoting Pekin ducks’ resistance to the virus at early stages. Meanwhile, interleukins have been described to mediate T and B lymphocyte activation, proliferation, and differentiation during immune activation and regulation [43,44,45]. Our results further demonstrate that the administration of pUC18-CpG with vaccines elicits higher IL-2 and IL-6 expression levels in ducks at 24 dpi, 35 dpi, and 14 dpi, respectively (Figure 3). IFN-γ and IL-2 are cytokines secreted by Th1 cells that mainly mediate cellular immune response, whereas IL-6 is a cytokine secreted by Th2 cells which mainly regulate humoral immune response, and the upregulation of these cytokines induces an enhanced Th1- and Th2- type response. These findings suggest that pUC18-CpG may stimulate the body continuously after immunization.

To further validate the immunostimulatory effects of pUC18-CpG, we also determined the efficacy of the different vaccines by assessing the proportion of ducks in each experimental group that had the live virus present in their serum following vaccination. We verified the presence of the virus in serum through RT-PCR methods. Although the traditional inactivated vaccine (group E and group G) reduced the incidence of morbidity and mortality in infected ducks, it was not capable of ultimately preventing infection. Alternatively, ducks vaccinated with CpG (groups A1, A2, B1, and B2) were found to be fully protected post-challenge, and the PD_50_ value of the pUC18-CpG adjuvant vaccines (A: 4.0; B: 4.5) was more than twice that of the traditional vaccine (E: 1.4; G: 1.8) (Table 3). Surprisingly, the PD_50_ in group G is higher than that in group E, which may be due to insufficient sample size and inevitable difference between individuals. Importantly, the control ducks exhibited clinical signs after receiving a challenge with virulent viruses, and tested positive by virus isolation from the serum of selected control ducks, confirming the validity of the protection criteria that we applied to our study.

Two limitations of this study need to be considered. First, groups vaccinated with a 1/2 or 1/4 dose should be also guaranteed 11 Pekin ducks for experiments. Second, the study just divided three vaccine doses for the calculation of PD_50_, whereas the dose of pUC18-CpG used was not optimized, but only referring previous publication. However, due to insufficient funds and isolators to support more grouping, we had to temporarily ignore these two issues.

Cumulatively, these results suggest that employing pUC18-CpG as an adjuvant induces a positive effect on the protective efficacy against DTMUV, and administration of a full dose of 1/2 DTMUV + 40 μg pUC18-CpG was the most commercial and effective.

## Figures and Tables

**Table 1:** Vaccine formulations.

Groups		Contents	Inactivated Viral Fluid Volume (mL)	pUC18-CpG (mg)	Aqueous Phase Volume (mL)	Oil Phase Volume (mL)	Vaccine Volume (mL)
A	A1	DTUMV + CpG	10	3.2	10	30	40
	A2		10	6.4	10	30	40
	A3		10	12.8	10	30	40
B	B1	1/2DTUMV + CpG	5	3.2	10	30	40
	B2		5	6.4	10	30	40
	B3		5	12.8	10	30	40
E	E1	DTUMV	10	0	10	30	40
	E2		10	0	10	30	40
	E3		10	0	10	30	40
G	G1	1/2DTUMV	5	0	10	30	40
	G2		5	0	10	30	40
	G3		5	0	10	30	40
I	I	PBS	/	/	/	/	40

The DTMUV-HB with a titer of approximately 10^7.5^ ELD_50_ (50% embryo lethal dose)/mL.

**Table 2:** Experimental design.

Groups		Contents	Inoculum Dose (mL)		Number of Ducks	Route of Immunizations	Times of Immunization	Purpose of the Experiment
A	A1	DTUMV + CpG	0.5	Full	11	IM	2	Immune responses to vaccine formulation X
	A2		0.25	1/2	6	IM	2	
	A3		0.125	1/4	6	IM	2	
B	B1	1/2DTUMV + CpG	0.5	Full	11	IM	2	
	B2		0.25	1/2	6	IM	2	
	B3		0.125	1/4	6	IM	2	
E	E1	DTUMV	0.5	Full	11	IM	2	
	E2		0.25	1/2	6	IM	2	
	E3		0.125	1/4	6	IM	2	
G	G1	1/2DTUMV	0.5	Full	11	IM	2	
	G2		0.25	1/2	6	IM	2	
	G3		0.125	1/4	6	IM	2	
I	I	PBS	/		12	IM	2	

According to the Manual of Diagnostic Tests and Vaccines for Terrestrial Animals issued by the World Organization for Animal Health, a commercial vaccine should have a PD_50_ (50% protection dose) value of at least 3 per dose. Each batch of vaccines, regardless of the immunization dose, guarantees that each duck has an immune dose of 40 μg of pUC18-CpG.

**Table 3:** PD_50_ value of each vaccine groups.

Groups		Dose	Protected/All	Protective Rate	PD_50_/0.5 mL
A	A1	Full	6/6	100%	4.0
	A2	1/2	6/6	100%	
	A3	1/4	3/6	50%	
B	B1	Full	6/6	100%	4.5
	B2	1/2	6/6	100%	
	B3	1/4	4/6	66.67%	
E	E1	Full	4/6	66.67%	1.4
	E2	1/2	1/6	16.67%	
	E3	1/4	1/6	16.67%	
G	G1	Full	4/6	66.67%	1.8
	G2	1/2	2/6	33.33%	
	G3	1/4	2/6	33.33%	

## Supplementary Materials

The following are available online at https://www.mdpi.com/1999-4915/12/2/238/s1, Figure S1: Serum hemagglutination inhibition (HI) levels in vaccinated ducks, Table S1: Results of HI assay and Virus challenge.
