# Supplementary material for: pUC18-CpG Is an Effective Adjuvant for a Duck Tembusu Virus Inactivated Vaccine
Source: Viruses. 2020 Feb 20;12(2):238. doi: 10.3390/v12020238 (PMC7077240; doi:10.3390/v12020238)
Supplement: Supplementary file 1 [file viruses-12-00238-s001.zip › viruses-706505-supplementary/Supplementary Materials-Figure S1.docx]

**Figure S1.** Serum hemagglutination inhibition (HI) levels in vaccinated ducks. Serum samples were collected at (A) 14-day after primary immunization, and at (B) 10, (C) 21, (D) 42-day after boost immunization for HI antibody level. Antibody titers were determined using the HI assay with 4 HA units of the DTMUV-HB. The HI titer is expressed as the reciprocal form. Data are expressed as means ± SEM. (ns: not significant)
